# THE CONFIDENCE FINANCIAL WELL-BEING INTERVENTION FOR LATINO FAMILY CAREGIVERS: PILOT STUDY RESULTS

**DOI:** 10.1093/geroni/igad104.1998

**Published:** 2023-12-21

**Authors:** Kylie Meyer, Susanna Mage, Donna Benton

## Abstract

Family caregivers to people living with dementia encounter an average of $10,697 per year in out-of-pocket caregiving costs that exert long-term material and psychological consequences. Latino caregivers encounter higher out-of-pocket caregiving costs relative to their income than non-Latino caregivers. There is an unmet need for programs to alleviate financial hardship among caregivers, particularly those culturally tailored to Latino caregivers. The Confidently Navigating Financial Decisions and Enhancing Financial Wellbeing in Dementia Caregiving (CONFIDENCE) program draws on successful intervention approaches identified within nursing and gerontological research to manage out-of-pocket caregiving costs and prevent financial hardship among caregivers to persons living with dementia. Between May 2022 and February 2023, CONFIDENCE was delivered to six cohorts of Latino caregivers at an academic-service center. This symposium will discuss preliminary pilot study results. Donna Benton, PhD, will describe how the intervention was developed with a community advisory council to ensure a culturally-tailored program. Kylie Meyer, PhD, will share the preliminary efficacy of the intervention based on interim analyses of outcomes, including caregiver resourcefulness and financial strain. Alexander Gonzales, BSG, will describe challenges related to recruitment and retention, and considers registration and fidelity data to understand possible causes of low attendance. Lastly, Susanna Mage, PhD(c), MA, will present the findings from the preliminary evaluation of the program to examine the acceptability, including participant satisfaction surveys and one-on-one interviews. Initial success of the pilot phase suggests opportunities for future efficacy testing. If efficacious, CONFIDENCE could help alleviate financial hardship experienced by family caregivers to persons living with dementia.

